# Study of muscle fibers of the extensor digitorium longus and soleus muscles of C57BL/6 females exposed to glyphosate during pregnancy and lactation

**DOI:** 10.31744/einstein_journal/2021AO5657

**Published:** 2021-08-02

**Authors:** Ariadne Barbosa, Mylena Campos Oliveira, Camila Kuhn-Fraga, Lucinéia de Fátima Chasko Ribeiro, Sandra Lucinei Balbo, Márcia Miranda Torrejais

**Affiliations:** 1 Universidade Estadual do Oeste do Paraná CascavelPR Brazil Universidade Estadual do Oeste do Paraná, Cascavel, PR, Brazil.

**Keywords:** Glyphosate, Pesticides, Toxicity, Maternal exposure, Muscle fibers, skeletal, Mice, inbred C57BL

## Abstract

**Objective:**

To evaluate the morphology and morphometry of the muscles extensor digitorium longus and soleus of C57BL/6 females, who were exposed to glyphosate during pregnancy and lactation.

**Methods:**

Twelve female mice from the C57BL/6 lineage were used. After detection of pregnancy, they were divided into a Control Group, which received only water, and a Glyphosate Group, which received water with 0.5% glyphosate during pregnancy and lactation. Both groups received *ad libitum* standard diet. After weaning, the females were euthanized and weighed; naso-anal length was measured, and fats were collected and weighed. The muscles extensor digitorium longus and soleus were collected, and their length and weight were measured. Then, the muscles were fixed in Methacarn to perform the histological study of muscle fibers.

**Results:**

Glyphosate Group presented lower weight gain during pregnancy and also lower final body weight and naso-anal length; however, the other body parameters evaluated did not present a significant difference in relation to the Control Group. Significant differences were also not observed in the analysis of muscle fibers and connective tissue.

**Conclusion:**

Exposure to 0.5% glyphosate during pregnancy and lactation resulted in lower weight gain during pregnancy, final weight, and naso-anal length. Despite not directly altering the morphology of muscle tissue, these results may indicate enough exposure to interfere with animal metabolism.

## INTRODUCTION

Glyphosate (N-(phosphonomethyl)glycine) is an organophosphorus compound that ranked first in the list of the ten best-selling active ingredients in Brazil, in 2018.^( 1 )^ It is present in the formulation of Roundup^®^Original DI (Monsanto do Brasil LTDA., São Paulo, SP, Brazil), one of the most widely used herbicides in the world,^( 2 )^which accounted for almost 72% of global pesticide use in 2016.^( 3 )^ Its mechanism of action consists in inhibiting the enzyme 5-enolpyruvylshikimate-3-phosphate synthase of the shikimate pathway, responsible for the production of the intermediate chorismate, a compound required in the synthesis of aromatic amino acids essential for plant development.^( 4 )^

Although this pathway is not present in mammals, studies have shown that the herbicide is toxic in rats^( 5 )^ and mice,^( 6 )^ as well as in humans,^( 7 )^ associated with the genesis of several diseases.^( 8 )^

In Brazil, there are still no limits set on glyphosate or any other herbicide in water or soil by regulatory agencies. According to the Environmental Protection Agency (EPA), an agency from the United States, the glyphosate limit in drinking water is 700µg/L, with an acceptable daily dose of 0.05mg/kg per body weight.^( 9 )^

However, it is common for the stipulated dose to be exceeded, which in turn, reflects in the increased concentration of this compound in the environment,^( 10 )^promoting contamination of rivers and surface waters,^( 11 )^ and becoming a potential source of exposure to humans.^( 12 )^ With regard to human health, studies have shown exposure to glyphosate has been recurrently associated with some health problems, such as cancer, endocrine disruption,^( 12 )^ depression, Parkinson’s disease, Alzheimer’s disease,^( 13 )^ among others.

In rodents, experimental studies have shown that pesticide exposure increases the incidence of tumors,^( 6 )^ and promotes abnormalities in liver, heart, and brain function,^( 14 )^ as well as damage to cell junctions of intestinal cells, leading to increased membrane permeability.^( 15 )^ Furthermore, organophosphate pesticides have been found to promote inhibition of acetylcholinesterase (AChE),^( 16 )^ and lead to degeneration^( 17 )^ and necrosis of muscle fibers.^( 18 )^

It is known that pregnancy is a period of numerous physiological changes, which promote the vulnerability of both mother and fetus. Therefore, it is strictly important to reduce exposure to any toxins during this period. However, maternal exposure to pesticides is becoming increasingly common, since it can occur through contact with air, water, contaminated food, in the work environment, during the mixing of chemical compounds, in the application of pesticides, in the cleaning of equipment, or even indirectly, during the handling of contaminated crops or food.^( 19 )^

Although some experimental studies report that exposure to glyphosate promotes changes in some tissues, and in the metabolism of the offspring of rats and mice,^( 2 , 20 )^the effects of exposure to this herbicide on the skeletal muscles of females exposed during pregnancy and lactation are not known yet. Thus, this study is of great importance for understanding of possible musculoskeletal changes promoted by exposure to glyphosate.

## OBJECTIVE

To evaluate the morphology and morphometry of the muscles extensor digitorum longus and soleus of C57BL/6 females exposed to glyphosate during pregnancy and lactation.

## METHODS

### Obtaining the animals

Initially, 30 C57BL/6 mice of reproductive age were used, 20 females and 10 males, aged between 60 and 90 days, with a mean body weight of 20g to 25g. The animals were kept under controlled temperature (28±2^o^C) and light conditions (12 hours light/dark), and received standard rodent chow (Supralab, São Leopoldo, RS, Brazil) and filtered water *ad libitum* throughout the experiment.

All experiments reported in this study were conducted in accordance with national and international legislation, as per the guidelines of the National Council for the Control of Animal Experimentation (CONCEA - *Conselho Nacional de Controle de Experimentação Animal* ) and the Public Health Service Policy on Humane Care and Use of Laboratory Animals (PHS Policy), under approval of the Ethics Committee on Animal Use of the *Universidade Estadual do Oeste do Paraná* (Unioeste), ordinance 3.730, of September 16, 2016, in the city of Cascavel (PR).

### Crossbreeding

After 7 days of acclimatization, vaginal smears were taken to follow the estrous cycle of females, which were allocated for mating when they were in proestrus, with the proportion of two females to one male, during the night. In the morning of the following day, the vaginal smear was taken again to identify spermatozoa and the estrous cycle was determined to confirm pregnancy. Females considered pregnant showed the presence of spermatozoa or a 4-day stay in the diestrous phase after mating. The females that were not pregnant were again submitted to the mating process until pregnancy was confirmed.

### Glyphosate administration

Once pregnancy was confirmed, the females were placed in individual boxes, separated into Control Group (CTL, n=6), which received filtered water during the entire period of pregnancy (21 days) and lactation (30 days), and Glyphosate Group (GF, n=6), which received the herbicide 0.5% glyphosate Roundup^®^ Original DI in drinking water, from the fourth day of pregnancy until the end of lactation. This dosage had been used in a previous study,^( 20 )^ and was chosen because it mimics direct groundwater contamination, because it is similar to the amount of pesticide found in water after agricultural practices.^( 21 )^The commercial formulation of Roundup^® ^Original DI glyphosate used contained 445g/L of N-(phosphonomethyl)glycine diammonium salt, equivalent to 370g/L (37.0%m/v) of the active component glyphosate [N-(phosphomethyl)glycine].

### Euthanasia of females

After 30 days of lactation, weaning occurred and the females were euthanized after completing two estrous cycles. The animals were anesthetized with xylazine hydrochloride (Anasedan^®^, Vetbrands, Axxon Group, Rio de Janeiro, RJ, Brazil) and ketamine hydrochloride (Dopalen^®^, Vetbrands, Axxon Group, Rio de Janeiro, RJ, Brazil) at concentrations of 9mg/kg and 90mg/kg, respectively, and were finally euthanized.

Females were weighed after euthanasia. Naso-anal length (NAL) was measured, and retroperitoneal and perigonadal fat was collected and weighed.

### Collection of the muscles extensor digitorum longus and soleus

To collect the extensor digitorum longus (EDL) muscle, the skin was detached and the tibialis anterior muscle was removed for dissection and removal of the EDL muscle. The gastrocnemius muscle was removed for dissection and removal of the soleus (SOL) muscle. The EDL and SOL muscles were weighed (g) on analytical scales (Shimadzu UX620H, São Paulo, SP, Brazil) and their length (mm) was measured with the aid of a digital pachymeter (Digimess^®^, São Paulo, SP, Brazil).

### Histological study

For the study of muscle fibers, the EDL and SOL muscles of the right antimere of the pelvic limbs were removed and stored in a glass container with Methacarn fixative. After 24 hours, they were transferred to 70% alcohol and embedded in paraffin, with an n-butyl alcohol embedding protocol.

From the analysis of ten microscopic fields (40x lens) for each animal, the EDL and SOL muscles were transversely cut and submitted to hematoxylin-eosin^( 22 )^staining, for morphological analysis of the muscle fibers, quantification of the numbers of nuclei and fibers, nucleus-fiber ratio, area, and major and minor diameter of each muscle fiber. The same cutting procedure was performed for Masson’s trichrome staining,^( 23 )^ which allows quantifying connective tissue, by analysis of ten microscopic fields for each animal (20x lens).

The images of the muscle fibers were obtained using an Olympus BX60^®^ microscope, coupled to an Olympus DP71 camera (Tokyo, Japan), with the aid of the DP Controler 3.2.1 276 software. The Image-Pro Plus 6.0^®^ software (Media Cybernetics, Maryland, USA) was used for morphological and morphometric analysis of the materials.

### Statistical analysis

The data obtained were submitted to statistical analysis using the GraphPad Prism^®^ (La Jolla, CA, USA) software, taking into consideration the results of the normality tests. For the data found to be normal, the statistical test used was the Student’s *t* test, whereas for the data that were not normal, the Mann-Whitney test was used. Values of p<0.05 were considered significant.

## RESULTS

### Pregnancy and lactation data

The GF Group had lower gestational weight gain (p=0.0327) when compared to the CTL Group ( Table 1 ). However, the data for weight loss during lactation, gestation time, and litter size showed no statistical differences when compared to the CTL Group ( Table 1 ). Body data

**Table 1:** Pregnancy and lactation data from mice in the Control and Glyphosate Groups

	Groups	
	CTL	GF
Weight gain during pregnancy, g	12±1.4	9.5±1.9*
Weight loss during lactation, g	1.8±1.9	1.3±2.4
Time of pregnancy, days	19±1.4	20±0.82
Litter size	6.7±1.6	5.0±1.5

Values expressed as mean±standard deviation.

* p<0.05. Student’s *t* test.

CTL: Control Group; GF: Glyphosate Group.

Animals from the GF Group showed lower body weight (p=0.0103) and NAL (p=0.0002) when compared to the CTL Group ( Table 2 ). In contrast, the parameters of retroperitoneal and perigonadal fat weights were similar between the Groups ( Table 2 ). The weight and length data of the EDL and SOL muscles also showed no statistical differences when comparing the Groups ( Table 2 ).

**Table 2:** Body parameters of mice in Control and Glyphosate Groups

Body parameters	Groups	
	CTL	GF
Final body weight, g	23±1.3	22±0.79*
NAL, cm	9.7±0.5	9.2±0.38*
Weight of fats, mg	469±42	602±173
EDL muscle weight, mg	7.2±1.8	10±4.9
EDL muscle length, mm	7.3±0.98	7.9±1.0
Soleus muscle weight, mg	6.1±0.81	6.2±2.2
Soleus muscle length, mm	6.1±1.3	6.3±1.2


Values expressed as mean±standard deviation.

* p<0.05. Student’s *t* test. Mann Whitney test.

CTL: Control Group; GF: Glyphosate Group; NAL: nasal-anal length; EDL: extensor digitorium longus.

### Morphological and morphometric analysis of muscle fibers

**Figure 1:**
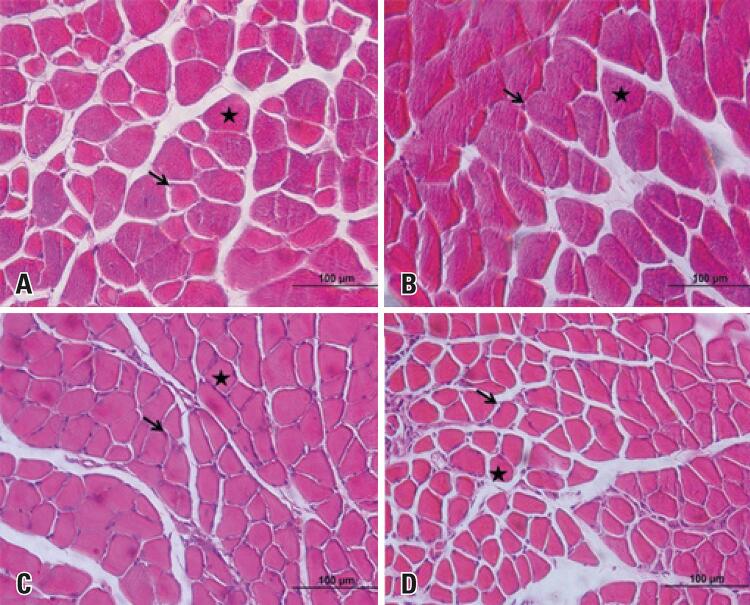
Photomicrographs of the extensor digitorum longus and soleus muscles of C57BL/6 mice from the Control and Glyphosate Groups. Cross-section, hematoxylin-eosin. Polygonal fibers (stars) and peripheral nuclei (arrows). (A) Extensor digitorum longus muscle from Control Group (A) and Glyphosate Group (B). Soleus muscle from Control Group (C) and Glyphosate Group (D)

The evaluation of the muscle fibers of the EDL and SOL muscles showed fibers with preserved morphology, maintaining the polygonal aspect, the presence of peripheral nuclei, and the eventual presence of central nuclei in the two groups studied ( Figure 1 ).

As to the morphometric analysis of the muscle fibers of the EDL and SOL muscles, none of the parameters evaluated showed significant differences between Groups CTL and GF ( Table 3 ).

**Table 3:** Morphometric analysis of the muscle fibers and connective tissue of the extensor digitorium longus muscle of mice in the Control and Glyphosate Groups

	EDL muscle		SOL muscle	
	CTL Group	GF Group	CTL Group	GF Group
Area, µm^2^	115±34	152±86	157±24	187±69
Major diameter, µm	15±1.8	17±4.8	18±1.4	19±3.1
Minor diameter, µm	9.6±1.4	11±3.2	11±0.83	12±2.7
Fiber density	536±131	578±243	414±123	430±175
Number of peripheral nuclei	617±109	707±195	927±237	827±265
Number of central nuclei	1.0±0.71	0.80±1.3	2.2±2.4	1.8±2.5
Nucleus/fiber ratio	1.2±0.16	1.3±0.22	2.3±0.34	1.9±0.15


Values expressed as mean±standard deviation. Student’s *t* test. Mann Whitney test.

EDL: extensor digitorum longus; SOL: soleus;

CTL: Control Group; GF: Glyphosate Group.

### Analysis of the amount of connective tissue

**Figure 2:**
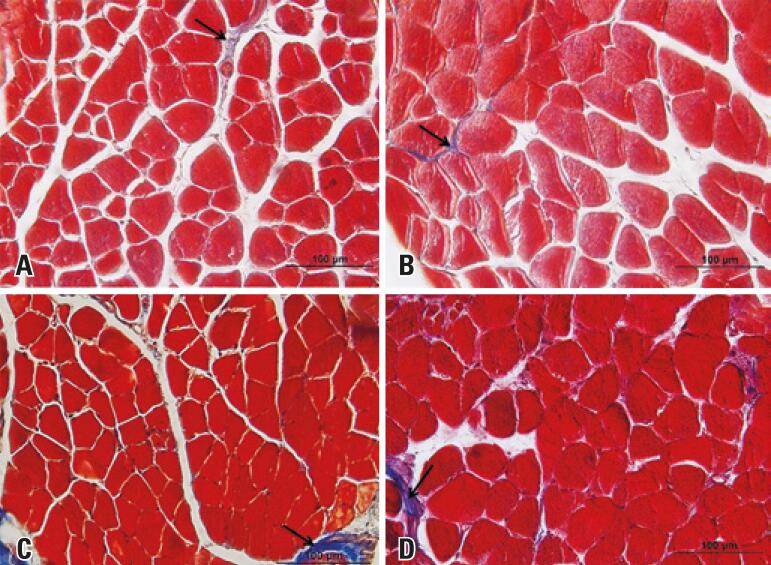
Photomicrographs of the extensor digitorum longus and soleus muscles from C57BL/6 mice of the Control and Glyphosate Groups. Cross-section stained with Masson’s trichrome. Connective tissue (arrows). Extensor digitorium longus muscle from Control (A) and Glyphosate (B) Groups. Soleus muscle from the Control (C) and Glyphosate (D) Groups

**Figure 3:**
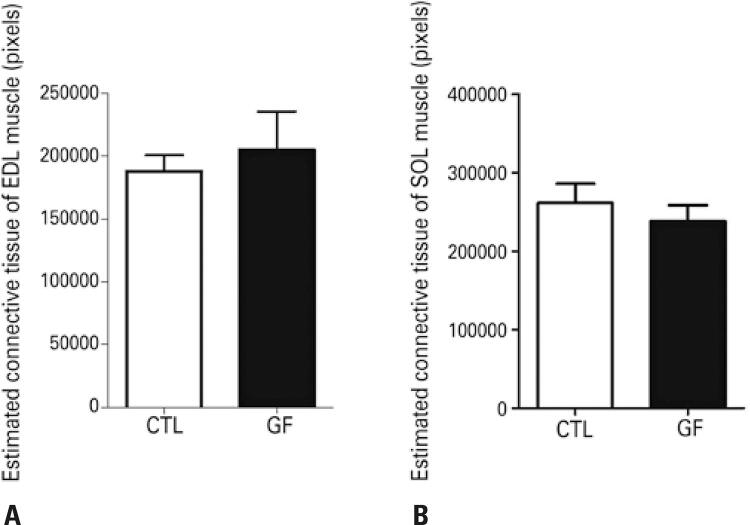
Graph of the estimated connective tissue found in the muscle fibers of the extensor digitorum longus and soleus muscles of C57BL/6 mice from the Control Group and Glyphosate Group, plotted in pixels

EDL: extensor digitorum longus muscle; SOL: soleus; CTL: Control Group; GF: Glyphosate Group.

Masson trichrome staining showed the presence of connective tissue between muscle fibers, especially in the perimysium involving the fascicles ( Figure 2 ). Regarding the estimated amount of connective tissue in the two muscles studied, no statistical differences were observed between the CTL and GF Groups ( Figure 3 ).

## DISCUSSION

The study showed the exposure of females to glyphosate during pregnancy and lactation promoted lower weight gain during gestation, which was also observed in other studies that exposed pregnant rats to 1%^( 14 , 24 )^ and 0.5%^( 2 )^ glyphosate concentrations. Furthermore, glyphosate exposure also resulted in lower final body weight and NAL of exposed animals, which corroborates the findings of Teleken et al.,^( 20 )^ who also exposed mice to 0.5% glyphosate during these phases of their life cycle.

Although the present study did not verify assess the animals’ water and food consumption, Beuret et al.,^( 24 )^and McKenna et al.,^( 25 )^showed animals exposed to glyphosate had lower water and food consumption compared to unexposed animals, which justifies the lower weight gain and the lower final body weight and NAL of the GF Group, since glyphosate administration may reflect in reduced palatability of ingested water, or promote changes in the thirst regulatory centers, due to the effects of the herbicide and its metabolites.^( 14 )^

As to muscle fibers, Bright et al.,^( 17 )^demonstrated that rats exposed to sublethal doses of sarin presented with degeneration in muscle fibers and mononuclear infiltrates in the diaphragm muscle, when euthanized 24 hours and 3 days after exposure, respectively. De Bleecker et al.,^( 18 )^noted that exposure to paraoxon compound promoted fiber necrosis in several muscle groups of rats, with predominance in the diaphragm muscle. However, mixed muscles, such as masseter and soleus, were also affected. In view of the results, the authors observed a correlation between the oxidative capacity of muscles and their susceptibility to necrosis, with mixed muscles showing a predominance of oxidative fibers being more prone to necrosis.

Although the literature findings demonstrated exposure to organophosphorus compounds promotes degeneration and necrosis of muscle fibers, as well as the relation between predominance of fiber type and susceptibility to necrosis, the same was not observed in the EDL and SOL muscle fibers upon exposure to glyphosate. This may be justified by recent findings showing the toxic potential of this herbicide during direct exposure is minimal, despite the current association of glyphosate exposure with occurrence of diseases.^( 26 )^ Thus, the absence of changes in the morphological and morphometric parameters of the EDL and SOL muscles of the females, and of any necrotic processes pointing to possible fiber degeneration, may be associated with the low toxicity of the herbicide in this first exposure.

However, even if the low toxicity of glyphosate in direct exposure is demonstrated, a study showed that, despite not causing effects in the first generation, this herbicide promotes an increase in the occurrence of diseases in the offspring of exposed rats. Hence, its ability to promote epigenetic changes, which will be transmitted to subsequent generations.^( 27 )^Due to the effects promoted by exposure, glyphosate has been investigated as a potential chemical endocrine disruptor,^( 28 )^ which consists of a substance capable of altering the maternal environment, and influencing the stages of intrauterine development, as well as increasing the risk of chronic diseases in adulthood.^( 29 )^

Despite low toxicity during direct exposure, the potential action of glyphosate as an endocrine disruptor can promote changes in exposed offspring, and it is strictly necessary to take this fact into account in the etiology of diseases in future generations.

## CONCLUSION

Exposure to 0.5% glyphosate during pregnancy and lactation promoted lower weight gain during gestation and lower body weight and size of females. Although the morphological characteristics of muscle tissue were not altered, the change in body parameters indicates glyphosate may interfere in the metabolism of the animal, promoting changes in its cycle of obtaining and storing energy.
