# Supplementary material for: Study of muscle fibers of the extensor digitorium longus and soleus muscles of C57BL/6 females exposed to glyphosate during pregnancy and lactation
Source: Einstein (Sao Paulo). 2021 Aug 2;19:eAO5657. doi: 10.31744/einstein_journal/2021AO5657 (PMC8328150; doi:10.31744/einstein_journal/2021AO5657)
Supplement: Translation

# Translation: Estudo das fibras musculares dos músculos extensor longo dos dedos e sóleo de fêmeas C57BL/6 expostas ao glifosato durante a prenhez e a lactação

**Authors:** Ariadne Barbosa, Mylena Campos Oliveira, Camila Kuhn-Fraga, Lucinéia de Fátima Chasko Ribeiro, Sandra Lucinei Balbo, Márcia Miranda Torrejais

**Affiliations:** 1 Brazil Universidade Estadual do Oeste do Paraná, Cascavel, PR, Brazil.

**Keywords:** Glifosato, Praguicidas, Toxicidade, Exposição materna, Fibras musculares esqueléticas, Camundongos endogâmicos C57BL

## Abstract

**Objetivo:**

Avaliar a morfologia e a morfometria dos músculos extensor longo dos dedos e sóleo de fêmeas C57BL/6 expostas ao glifosato durante a prenhez e lactação.

**Métodos:**

Foram utilizados 12 camundongos fêmeas da linhagem C57BL/6. Após detecção da prenhez, foram separadas em Grupo Controle, que recebeu somente água, e Grupo Glifosato, que recebeu água com 0,5% de glifosato durante a prenhez e lactação. Ambos os grupos receberam dieta padrão *ad libitum* . Após o desmame, as fêmeas foram eutanasiadas e pesadas; o comprimento nasoanal foi mensurado, e as gorduras foram coletadas e pesadas. Os músculos extensor longo dos dedos e sóleo foram coletados, e seu comprimento e peso foram mensurados. Em seguida, os músculos foram fixados em *Methacarn* para a realização do estudo histológico das fibras musculares.

**Resultados:**

O Grupo Glifosato apresentou menor ganho de peso durante a prenhez e também menor peso corporal final e comprimento nasoanal, entretanto os demais parâmetros corporais avaliados não apresentaram diferença significativa em relação ao Grupo Controle. Na análise das fibras musculares e do tecido conjuntivo, também não foram observadas diferenças significativas.

**Conclusão:**

A exposição a 0,5% de glifosato durante a prenhez e lactação resultou em menor ganho de peso na gestação, peso final e comprimento nasoanal, o que pode indicar que, apesar de não alterar a morfologia do tecido muscular diretamente, a exposição foi suficiente para interferir no metabolismo dos animais.

## INTRODUÇÃO

O glifosato (N-(fosfonometil)glicina) é um composto organofosforado que ocupou o primeiro lugar na lista dos dez ingredientes ativos mais vendidos no Brasil, em 2018.^( 1 )^Está presente na formulação do Roundup^®^Original DI (Monsanto do Brasil LTDA., São Paulo, SP, Brasil), um dos herbicidas mais utilizados no mundo,^( 2 )^que correspondeu a quase 72% do uso global de pesticidas em 2016.^( 3 )^Seu mecanismo de ação consiste na inibição da enzima 5-enolpiruvilchiquimato-3-fosfato sintase da via do chiquimato, responsável pela produção do intermediário corismato, composto necessário na síntese dos aminoácidos aromáticos essenciais para o desenvolvimento da planta.^( 4 )^

Embora essa via não esteja presente nos mamíferos, estudos já demonstraram que o herbicida possui toxicidade em ratos^( 5 )^e camundongos,^( 6 )^bem como em humanos,^( 7 )^estando associado à gênese de diversas doenças.^( 8 )^

No Brasil, ainda não há limite estabelecido de glifosato ou qualquer outro herbicida na água ou solo pelas agências controladoras. De acordo com a *Environmental Protection Agency* (EPA), agência norte-americana, o limite de glifosato em água potável compreende 700µg/L, com dose diária aceitável de 0,05mg/kg por massa corpórea.^( 9 )^

É, porém, comum que a dose estipulada seja ultrapassada, o que, por sua vez, reflete no aumento da concentração desse composto no ambiente,^( 10 )^promovendo a contaminação de rios e águas superficiais,^( 11 )^tornando-se, assim, uma potencial fonte de exposição a seres humanos.^( 12 )^Com relação à saúde humana, estudos demonstram que a exposição ao glifosato tem sido recorrentemente associada a problemas de saúde, como câncer, desregulação endócrina,^( 12 )^depressão, mal de Parkinson, doença de Alzheimer,^( 13 )^dentre outros.

Em roedores, estudos experimentais demonstraram que a exposição a pesticidas aumenta a incidência de tumores^( 6 )^e promove anormalidades no funcionamento do fígado, coração e cérebro,^( 14 )^assim como danos nas junções celulares de células intestinais, que levaram à maior permeabilidade de membrana.^( 15 )^Ainda, foi observado que pesticidas organofosforados promovem a inibição da enzima acetilcolinesterase (ACchE)^( 16 )^e levam à degeneração^( 17 )^e à necrose das fibras musculares.^( 18 )^

Sabe-se que a gestação é um período de numerosas alterações fisiológicas, o que promove a vulnerabilidade, tanto da mãe quanto do feto. Dessa forma, é estritamente importante a redução da exposição a quaisquer toxinas nesse período. Entretanto, a exposição materna a pesticidas torna-se cada vez mais comum, uma vez que pode ocorrer por meio do contato com o ar, a água, a comida contaminada, no ambiente de trabalho, durante a mistura de compostos químicos, na aplicação de pesticidas, na limpeza de equipamentos ou até de maneira indireta, durante o manuseio de plantações ou alimentos contaminados.^( 19 )^

Embora alguns estudos experimentais relatem que a exposição ao glifosato promova alterações em alguns tecidos, e no metabolismo da prole de ratos e camundongos,^( 2 , 20 )^ainda não são conhecidos os efeitos da exposição a esse herbicida na musculatura esquelética de fêmeas expostas durante a prenhez e lactação. Com isso, a realização deste estudo torna-se de grande importância para o entendimento das possíveis alterações musculoesqueléticas promovidas pela exposição ao glifosato.

## OBJETIVO

Avaliar a morfologia e a morfometria dos músculos extensor longo dos dedos e sóleo de fêmeas C57BL/6 expostas ao glifosato durante a prenhez e lactação.

## MÉTODOS

### Obtenção dos animais

Inicialmente, foram utilizados 30 camundongos C57BL/6 em idade reprodutiva, sendo 20 fêmeas e dez machos, com idade entre 60 e 90 dias de vida, e média de peso corporal de 20g a 25g. Os animais foram mantidos em condições de temperatura (28±2^o^C) e luminosidade (12 horas claro/escuro) controladas, e receberam ração padrão para roedores (Supralab, São Leopoldo, RS, Brasil) e água filtrada *ad libitum* durante todo o experimento.

Todas as experiências relatadas neste estudo foram conduzidas de acordo com a legislação nacional e internacional, norteadas pelas diretrizes do Conselho Nacional de Controle de Experimentação Animal (CONCEA) e pela *Public Health Service Policy on Humane Care and Use of Laboratory Animals* (PHS Policy), sob aprovação do Comitê de Ética em Uso de Animais da Universidade Estadual do Oeste do Paraná (Unioeste), portaria 3.730, de 16 de setembro de 2016, em Cascavel (PR).

### Cruzamentos

Passados 7 dias de aclimatação, foi realizado o esfregaço vaginal para acompanhamento do ciclo estral das fêmeas, que foram alocadas para realização dos cruzamentos quando se encontravam em fase de proestro, na proporção de duas fêmeas para um macho, durante o período noturno. Na manhã do dia seguinte, foi realizado novamente o esfregaço vaginal, para identificação de espermatozoides, e foi feita determinação do ciclo estral, para confirmação da prenhez. As fêmeas consideradas prenhas apresentaram presença de espermatozoides ou permanência de 4 dias na fase de diestro após o cruzamento. As fêmeas que não estavam prenhas passavam novamente pelo processo de cruzamento, até a confirmação da prenhez.

### Administração do glifosato

Confirmada a prenhez, as fêmeas foram alocadas em caixas individuais, sendo separadas em Grupo Controle (CTL, n=6), que recebeu água filtrada durante todo o período de prenhez (21 dias) e lactação (30 dias), e Grupo Glifosato (GF, n=6), que recebeu a adição de 0,5% do herbicida glifosato Roundup^®^Original DI na água de beber, a partir do quarto dia de prenhez até o fim da lactação. Essa dosagem já tinha sido utilizada em estudo prévio,^( 20 )^e foi escolhida por mimetizar a contaminação direta de águas subterrâneas, uma vez que se aproxima da quantidade de pesticida encontrada na água após práticas agrícolas.^( 21 )^A fórmula comercial do glifosato Roundup^®^Original DI utilizada continha 445g/L de sal de diamônio de N-(fosfonometil)glicina, equivalentes a 370g/L (37,0%m/v) do componente ativo glifosato N-(fosfometil)glicina.

### Eutanásia das fêmeas

Após 30 dias de amamentação, ocorreu o desmame, e a eutanásia das fêmeas foi realizada, depois de completados dois ciclos estrais. Os animais foram anestesiados com cloridrato de xilazina (Anasedan^®^, Vetbrands, Axxon Group, Rio de Janeiro, RJ, Brasil) e cloridrato de cetamina (Dopalen^®^, Vetbrands, Axxon Group, Rio de Janeiro, RJ, Brasil) em concentração de 9mg/kg e 90mg/kg, respectivamente, tendo sidos, por fim, eutanasiados.

Após a eutanásia, as fêmeas foram pesadas. O comprimento nasoanal (CNA) foi mensurado, bem como foram coletadas e pesadas as gorduras retroperitoneal e perigonadal.

### Coleta dos músculos extensor longo dos dedos e sóleo

Para a coleta do músculo extensor longo dos dedos (ELD), a pele foi rebatida, e o músculo tibial anterior foi removido, para a dissecção e a remoção do músculo ELD. Já o músculo gastrocnêmio foi retirado para a dissecção e a remoção do músculo sóleo (SOL). Os músculos ELD e SOL foram pesados (g) em balança analítica (Shimadzu UX620H, São Paulo, SP, Brasil) e seu comprimento (mm) foi mensurado com o auxílio de paquímetro digital (Digimess^®^, São Paulo, SP, Brasil).

### Estudo histológico

Para o estudo das fibras musculares, os músculos ELD e SOL do antímero direito dos membros pélvicos foram retirados e armazenados em recipiente de vidro com fixador Methacarn. Após 24 horas, foram transferidos para álcool 70% e incluídos em parafina, com protocolo de emblocamento em álcool n-butílico.

Os músculos ELD e SOL foram seccionados transversalmente e submetidos à coloração de hematoxilina-eosina,^( 22 )^para a análise morfológica das fibras musculares, quantificação dos números de núcleos e fibras, relação núcleo e fibra, área, diâmetro maior e diâmetro menor de cada fibra muscular, a partir da análise de dez campos microscópicos (objetiva de 40x) para cada animal. O mesmo procedimento de corte foi realizado para coloração de tricrômico de Masson,^( 23 )^que permite a quantificação do tecido conjuntivo, por meio da análise de dez campos microscópicos para cada animal (objetiva de 20x).

As imagens das fibras musculares foram obtidas por meio do microscópio Olympus BX60^®^acoplado à câmera Olympus DP71 (Tóquio, Japão), com auxílio do programa DP Controler 3.2.1 276. Foi utilizado o programa Image-Pro Plus 6.0^®^(Media Cybernetics, Maryland, Estados Unidos) para a análise morfológica e morfométrica dos materiais.

### Análise estatística

Os dados obtidos foram submetidos à análise estatística utilizando o programa GraphPad Prism^®^(La Jolla, CA, Estados Unidos), levando-se em consideração os resultados dos testes de normalidade. Para os dados encontrados em normalidade, o teste estatístico empregado foi o teste *t* de Student, enquanto para os dados que não estavam em normalidade, foi utilizado o teste de Mann-Whitney. Foram considerados significativos valores de p<0,05.

## RESULTADOS

### Dados do período de prenhez e lactação

O Grupo GF apresentou menor ganho de peso durante a gestação (p=0,0327) quando comparado ao Grupo CTL ( Tabela 1 ). Porém, os dados relativos à perda de peso durante lactação, ao tempo de gestação e ao tamanho da ninhada não apresentaram diferenças estatísticas quando comparados aos do Grupo CTL ( Tabela 1 ).

**Tabela 1:** Dados do período de prenhez e lactação de camundongos dos Grupos Controle e Glifosato

	Grupos	
	CTL	GF
Ganho de peso gestação, g	12±1,4	9,5±1,9*
Perda de peso lactação, g	1,8±1,9	1,3±2,4
Tempo de gestação, dias	19±1,4	20±0,82
Tamanho da ninhada	6,7±1,6	5,0±1,5

Valores expressos como média±desvio padrão.

* p<0,05. Teste *t* de Student.

CTL: Grupo Controle; GF: Grupo Glifosato.

### Dados corporais

Os animais do Grupo GF apresentaram menor peso corporal (p=0,0103) e CNA (p=0,0002) quando comparados aos do Grupo CTL ( Tabela 2 ). Em contrapartida, os parâmetros de pesos das gorduras retroperitoneal e perigonadal foram semelhantes entre os grupos ( Tabela 2 ). Os dados de peso e comprimento dos músculos ELD e SOL também não apresentaram diferenças estatísticas quando realizada a comparação entre os grupos ( Tabela 2 ).

**Tabela 2:** Parâmetros corporais de camundongos dos Grupos Controle e Glifosato

Parâmetros corporais	Grupos	
	CTL	GF
Peso corporal final, g	23±1,3	22±0,79*
CNA, cm	9,7±0,5	9,2±0,38*
Peso das gorduras, mg	469±42	602±173
Peso do músculo ELD, mg	7,2±1,8	10±4,9
Comprimento do músculo ELD, mm	7,3±0,98	7,9±1,0
Peso do músculo sóleo, mg	6,1±0,81	6,2±2,2
Comprimento do músculo sóleo, mm	6,1±1,3	6,3±1,2

Valores expressos como média±desvio padrão.

* p<0,05. Teste *t* de Student. Teste Mann Whitney.

CTL: Grupo Controle; GF: Grupo Glifosato; CNA: comprimento nasoanal; ELD: extensor longo dos dedos.

### Análise morfológica e morfométrica das fibras musculares

A avaliação das fibras musculares dos músculos ELD e SOL demonstrou fibras com morfologia preservada, mantendo o aspecto poligonal, a presença de núcleos periféricos e a eventual presença de núcleos centrais nos dois grupos estudados ( Figura 1 ).

**Figura 1:** Fotomicrografias dos músculos extensor longo dos dedos e sóleo de camundongos C57BL/6 dos Grupos Controle e Glifosato. Corte transversal, hematoxilina-eosina. Fibras poligonais (estrelas) e núcleos periféricos (setas). (A) Músculo extensor longo dos dedos do Grupo Controle (A) e do Grupo Glifosato (B). Músculo sóleo do Grupo Controle (C) e do Grupo Glifosato (D)

Quanto à análise morfométrica das fibras musculares dos músculos ELD e SOL, nenhum dos parâmetros avaliados apresentou diferenças significativas entre os Grupos CTL e GF ( Tabela 3 ).

**Tabela 3:** Análise morfométrica das fibras musculares e tecido conjuntivo dos músculos extensor longo dos dedos e sóleo de camundongos dos Grupos Controle e Glifosato

	Músculo ELD		Músculo SOL	
	Grupo CTL	Grupo GF	Grupo CTL	Grupo GF
Área, µm^2^	115±34	152±86	157±24	187±69
Diâmetro maior, µm	15±1,8	17±4,8	18±1,4	19±3,1
Diâmetro menor, µm	9,6±1,4	11±3,2	11±0,83	12±2,7
Densidade de fibras	536±131	578±243	414±123	430±175
Número de núcleos periféricos	617±109	707±195	927±237	827±265
Número de núcleos centrais	1,0±0,71	0,80±1,3	2,2±2,4	1,8±2,5
Relação núcleo/fibra	1,2±0,16	1,3±0,22	2,3±0,34	1,9±0,15

Valores expressos como média±desvio padrão.

Teste *t* de Student. Teste de Mann-Whitney.

ELD: extensor longo dos dedos; SOL: sóleo; CTL: Grupo Controle; GF: Grupo Glifosato.

### Análise da quantidade de tecido conjuntivo

A coloração com tricrômico de Masson evidenciou a presença de tecido conjuntivo entre as fibras musculares, principalmente no perimísio envolvendo os fascículos ( Figura 2 ). Quanto à estimativa da quantidade de tecido conjuntivo nos dois músculos estudados, não foram observadas diferenças estatísticas entre os Grupos CTL e GF ( Figura 3 ).

**Figura 2:** Fotomicrografias dos músculos extensor longo dos dedos e sóleo de camundongos C57BL/6 dos Grupos Controle e Glifosato. Corte transversal corado com tricrômico de Masson. Tecido conjuntivo (setas). Músculo extensor longo dos dedos do Grupo Controle (A) e do Grupo Glifosato (B). Músculo sóleo do Grupo Controle (C) e do Grupo Glifosato (D)

**Figura 3:** Gráfico da estimativa de tecido conjuntivo encontrado nas fibras musculares dos músculos extensor longo dos dedos e sóleo de camundongos C57BL/6, dos Grupo Controle e Grupo Glifosato, representado em *pixels*

ELD: extensor longo dos dedos; SOL: sóleo; CTL: Grupo Controle; GF: Grupo Glifosato.

## DISCUSSÃO

O estudo demonstrou que a exposição das fêmeas ao glifosato durante o período de prenhez e lactação promoveu menor ganho de peso na gestação, o que também foi observado em outros trabalhos que expuseram ratas prenhas às concentrações de 1%^( 14 , 24 )^e 0,5%^( 2 )^de glifosato. Além disso, a exposição ao glifosato também resultou em menor peso corporal final e CNA dos animais expostos, o que corrobora os achados de Teleken et al.,^( 20 )^que também expuseram camundongos à concentração de 0,5% de glifosato durante essas fases de seu ciclo de vida.

Embora o presente estudo não tenha verificado realizado o consumo hídrico e alimentar dos animais, Beuret et al.,^( 24 )^e McKenna et al.,^( 25 )^demonstram que animais expostos ao glifosato apresentaram menor consumo hídrico e alimentar comparados aos não expostos, o que justifica o menor ganho de peso e os menores peso corporal final e CNA do Grupo GF, uma vez que a administração do glifosato pode refletir na redução da palatabilidade da água ingerida, ou promover alterações nos centros reguladores da sede, devido aos efeitos do herbicida e seus metabólitos.^( 14 )^

Quanto às fibras musculares, Bright et al.,^( 17 )^demonstraram que ratos expostos a doses subletais de sarin apresentaram degeneração nas fibras musculares e infiltrado mononuclear no músculo diafragma, quando eutanasiados 24 horas e 3 dias após a exposição, respectivamente. Já De Bleecker et al.,^( 18 )^notaram que a exposição ao composto paraoxon promoveu necrose nas fibras de diversos grupos musculares de ratos, com predominância no músculo diafragma. Entretanto, músculos mistos, como o masseter e o SOL, também foram afetados. Diante dos resultados, os autores observaram correlação entre a capacidade oxidativa dos músculos e sua suscetibilidade à necrose, sendo os músculos mistos com predominância de fibras oxidativas mais propensos à necrose.

Embora os achados da literatura demonstrem que a exposição a compostos organofosforados promovam a degeneração e a necrose das fibras musculares, bem como a relação entre predominância do tipo de fibra e suscetibilidade à necrose, o mesmo não foi observado nas fibras dos músculos ELD e SOL na exposição ao glifosato. Isso pode ser justificado por achados recentes, segundo os quais o potencial tóxico desse herbicida durante a exposição direta é mínimo, apesar da corrente associação da exposição ao glifosato com a ocorrência de doenças.^( 26 )^Assim, as ausências de alterações nos parâmetros morfológicos e morfométricos dos músculos ELD e SOL das fêmeas, e de quaisquer processos necróticos que apontem possíveis degeneração das fibras, podem estar associadas à baixa toxicidade do herbicida nessa primeira exposição.

Entretanto, mesmo que demonstrada a baixa toxicidade do glifosato na exposição direta, estudo demonstra que, apesar de não causar efeitos na primeira geração, esse herbicida promove aumento na ocorrência de doenças nos descendentes dos ratos expostos. Daí sua capacidade de promover alterações epigenéticas, que serão transmitidas para as gerações seguintes.^( 27 )^Devido aos efeitos promovidos pela exposição, o glifosato tem sido investigado como potencial disruptor endócrino químico,^( 28 )^que consiste em uma substância capaz de alterar o ambiente materno, e influenciar na trajetória do desenvolvimento intrauterino, bem como aumentar o risco de doenças crônicas na vida adulta.^( 29 )^

Apesar de apresentar baixa toxicidade durante a exposição direta, a potencial ação do glifosato como disruptor endócrino pode promover alterações nos descendentes expostos, sendo estritamente necessário levar tal fato em consideração na etiologia das doenças nas futuras gerações.

## CONCLUSÃO

Exposição a 0,5% de glifosato, durante a prenhez e lactação, promoveu menor ganho de peso durante a gestação e menores peso e tamanho corporal das fêmeas. Embora as características morfológicas do tecido muscular não tenham sido alteradas, a mudança nos parâmetros corporais indica que o glifosato pode interferir no metabolismo do animal, promovendo alterações em seu ciclo de obtenção e reserva de energia.
